# Advances in long noncoding RNAs: identification, structure prediction and function annotation

**DOI:** 10.1093/bfgp/elv022

**Published:** 2015-06-13

**Authors:** Xingli Guo, Lin Gao, Yu Wang, David K. Y. Chiu, Tong Wang, Yue Deng

**Keywords:** lncRNA, identification, protein-coding potential, secondary structure prediction, function annotation

## Abstract

Long noncoding RNAs (lncRNAs), generally longer than 200 nucleotides and with poor protein coding potential, are usually considered collectively as a heterogeneous class of RNAs. Recently, an increasing number of studies have shown that lncRNAs can involve in various critical biological processes and a number of complex human diseases. Not only the primary sequences of many lncRNAs are directly interrelated to a specific functional role, strong evidence suggests that their secondary structures are even more interrelated to their known functions. As functional molecules, lncRNAs have become more and more relevant to many researchers. Here, we review recent, state-of-the-art advances in the three levels (the primary sequence, the secondary structure and the function annotation) of the lncRNA research, as well as computational methods for lncRNA data analysis.

## Introduction

While <2% of the human genome has been reported as protein-coding regions (∼20 000 genes) [1, 2], a large part of the genome gives rise to noncoding RNAs (ncRNAs), which have little or no protein-coding capability [3, 4]. Even though many classes of short ncRNAs, such as microRNAs (miRNA) and Piwi-interacting RNAs [5, 6] are widely studied, heterogeneous ncRNAs with length longer than 200 nucleotides (called long noncoding RNAs or lncRNAs) attract extensive interests from researchers [7]. With the rapid progress in high-throughput sequencing technology, thousands of lncRNAs have been identified in the mammals [8].

A hypothesis is that most currently annotated lncRNAs are not functional [9] and there are two reasons supporting this point. One is that like all biochemical processes, the transcription machinery is not perfect and can produce spurious RNAs that have no significant biological purpose [10], albeit many lncRNAs would be capped, spliced and polyadenylated just like mRNAs, none of these features offer informative indicator of function. The other is that even though the act of transcription matters, the product of transcription does not [9]. These would include RNAs generated during transcriptional interference, which involves the transcription of noncoding loci that overlaps the regulatory regions and is known to regulate gene expression in both prokaryotes and eukaryotes [11]. However, more and more lncRNAs are reported to play critical roles in biological processes. For example, the *Xist* RNA, which is required for mammalian dosage compensation [12], is clearly functional. And the roster of biological events in which lncRNAs are key factors is rapidly growing. These events include cell-cycle regulation, apoptosis, establishment of cell identity [13–15] as well as others. More importantly, dysregulation of lncRNAs is associated with a variety of human diseases, including cancer and other immune and neurological disorders [16–18]. As lncRNAs are crucial regulators of gene expression, it is expected that their dysregulation will lead to abnormal cellular function, growth defects and many human diseases. Analysis of the expression profiles of lncRNAs in a variety of cancer cells, and their comparison with that in corresponding normal cells, demonstrated that many lncRNAs are dysregulated in a wide range of cancers [18]. Furthermore, multiple lines of evidence increasingly link mutations and dysregulations of lncRNAs to diverse human diseases [19, 20]. Alterations in the primary structure, secondary structure and expression levels of lncRNAs as well as their cognate RNA-binding proteins underlie diseases ranging from neurodegeneration to cancers [21]. As for the process of cancer metastasis, which consists of a series of sequential and complex steps, lncRNAs exhibit distinct gene expression patterns in primary tumors and metastases, which can be used for cancer diagnosis and prognosis and served as potential therapeutic targets [22].

Even though lncRNAs have attracted increasing research interests, specific features in their sequences, secondary structures and the functional mechanisms for most lncRNAs remain unknown. The aim of this article is to bring together the scattered findings in lncRNA studies, focusing on the three levels relating sequence, structure and function. The lncRNA-related resources are also provided. We believe that this review will enable researchers to understand the key issues, and facilitate further advances in understanding the lncRNAs.

## Basic features In the lncRNA sequences and their identification

Unfortunately at present with our limited knowledge, there is no clear positive definition for lncRNAs. Generally, lncRNAs are still loosely defined as RNA transcripts more than 200 nucleotides (nt) long that can not be translated into a protein [23]. Nonetheless, the basic features of lncRNAs can be comparable with mRNAs, which can be translated into proteins. First, the size and the exons in lncRNAs are considered. In a set of human annotated lincRNAs (long intergenic ncRNAs, a subset of lncRNAs) [24], the average size of these lincRNAs are found to be smaller than that of mRNAs. They have fewer exons on average, which may partly be attributed to both the lower abundance and the incomplete assembly. It has been reported that lncRNAs have an unusual exonic structure, but exhibit standard canonical splice site signals and alternative splicing [25]. In the data set from Cabili *et al*. [24], most lncRNAs are spliced (98%) and show a striking tendency to have only two exons (42% of lncRNA transcripts versus 6% of mRNAs). Second, similar to mRNAs, many lncRNAs are characterized by ‘K4–K36’ domains, which consist of histone 3 Lys 4 trimethylation at the promoter followed by histone 3 Lys 36 trimethylation along the transcribed region [8, 24–26]. Third, there is substantial evidence to indicate that lncRNAs, just like mRNAs, are transcribed by RNA polymerase II and usually contain canonical polyadenylation signals, even though it is found that some lncRNAs are likely to be transcribed by polymerase III [27]. Fourth, generally unlike protein-coding genes, which are usually conserved across the species, most lncRNAs are poorly conserved, and thus have been taken for transcriptional noise [28]. Even though lncRNAs are less conserved than mRNAs in most cases, this by itself does not necessarily mean a lack of functionality. Generally lncRNA promoters are more conserved than their exons, and even as conserved as the mRNA promoters [24–26]. Previous evidence has reported that purifying selection exists in different sets of lncRNAs [26, 29, 30]. The expressed orthologs of a few highly conserved and brain-expressed mouse lncRNAs have also been identified in species as distant as opossums and chickens [24]. Although lncRNAs have low sequence conservation [31, 32], increasing evidence indicates critical roles played by lncRNAs, which will be illustrated later in this review.

Transcription of lncRNAs was first observed with traditional cloning methods without any further detection of translation products [33], such as *H19* [34]. A major progress in experimental identification of lncRNAs came with microarrays and tilling arrays, and more recently with next-generation sequencing technologies [4, 35, 36]. It was the FANTOM project [4, 35] in which cDNA cloning followed by Sanger sequencing that identified >34 000 lncRNAs in different mouse tissues. A significant portion of these lncRNAs had confident support [37, 38]. For example, lncRNAs identified in the GENCODE V7 [25] and the current RefSeq issue [39] were based on the refined EST and cDNA data. A special method of screening chromatin signatures such as ‘K4–K36’ domain had identified several thousands of lincRNAs in mouse and human [8, 26].

It was in recent years that thousands of lncRNAs have been identified owing to the broad applications of next-generation sequencing technologies [24, 40, 41]. It is worth mentioning that methods based on the next-generation sequencing data have discovered dozens of lncRNAs expressed in various samples of cancer cells and cell types. Furthermore, a canonical classification method has been applied [13, 17, 25] to categorize lncRNAs, by which lncRNAs have been grouped into five biotypes according to their proximity to protein-coding genes: sense, antisense, bidirectional, intronic and intergenic. Regarding the fact that some transcripts can have both coding and noncoding functions [42], Ulitsky *et al.* [9] have discussed the complexity of classification of noncoding transcripts and with examples.

Indeed, determining the protein coding ability for a transcript is critical in the identification of lncRNAs. It is also challenging because an lncRNA is likely to contain a putative open reading frame (ORF) purely by chance [42]. Accordingly, the principles such as a lack of evolutionary conservation of the identified ORFs, a lack of homology to known protein domains and a lack of the ability to template significant protein production [34, 43] have been generalized to distinguish coding potential across thousands of transcripts. Several recent methods and the measures used by them are described in Table 1 . The method of scoring conserved ORFs across dozens of species is used in [44, 45], which used the ‘codon substitution frequency’ to develop algorithms to score conserved ORFs across dozens of species, and provide a general strategy for determining the coding potential. But conservation-based methods may fail to detect young proteins because they do not contain a conserved ORF [44, 45]. Searching for a putative ORF and a homology in a large protein-domain database Pfam [50] is employed by a tool called Coding Potential Calculator (CPC) [46]. Another method named Coding-Potential Assessment Tool [47], similar to CPC, employed the information of ORF embedded in transcripts to develop the classifier. Different from previous works, Sun *et al.* [48] proposed a method to classify protein-coding and lncRNA transcripts by exploiting the intrinsic components contained in sequences instead of predicting the ORF. Additionally, CONC [49] is developed and applied in the FANTOM project, and another gene identification program GeneID [51] is used to measure the protein coding potential for lncRNAs in GENCODE v7. Sequencing RNAs associated with polyribosomes is used in the experimental method of [52], in which ribosome profiling has provided a strategy for identifying the ribosome occupancy on RNAs to distinguish the coding and noncoding transcripts.

**Table 1:** Tools for calculating the protein coding potential

Name	Features	Classifier	Reference
CSF Coding Substitution Frequency	Log-likelihood ratio of coding versus noncoding based on empirical frequencies of all codon substitutions in combination with evolutionary information for several organisms	Heuristic strategy	[44]
PhyloCSF	A rigorous reformulation of CSF features	Statistical models; https://github.com/mlin/PhyloCSF/wiki	[45]
CPC Coding Potential Calculator	Three features based on identified ORF and three features based on the output of parsing protein database	SVM classifier; http://cpc.cbi.pku.edu.cn/	[46]
CPAT Coding-Potential Assessment Tool	Four features based on ORF	Logistic regression model; http://lilab.research.bcm.edu/cpat/	[47]
CNCI Coding–Noncoding Index	Five features based on usage frequency of adjoining nucleotide triplets	SVM classifier; http://www.bioinfo.org/software/cnci/	[48]
CONC Coding or Noncoding	Nine features based on sequence composition, secondary structure and alignment with proteins	SVM classifier	[49]

## Probing lncRNA secondary structures

It is acknowledged that the secondary structure plays an important role for most ncRNA classes, including some lncRNAs [53–56]. Despite the prevalence of their secondary structure-mediated roles, the secondary structures for many lncRNAs in relation to their functions remain largely unknown. Here we describe some recent progresses related to the lncRNA secondary structures.

In general, the RNA secondary structure plays many key roles in molecular biology, more so than the primary sequence. The characteristics of the lncRNA secondary structure have occupied researchers and clinicians recently. For example, in the functional investigation of lncRNA *MALAT1*, it was reported that *MALAT1* clearly has a fascinating tRNA-like structure at its 3′ end [57, 58]. Another example is the steroid receptor RNA activator (*SRA*), which is 0.87 kb in length, and is organized into four domains with various secondary structure elements ranging from small, autonomous helical stems to larger structures formed via long-range base pairing [55]. *SPRY4-IT1* (AK024556), which is a cancer-associated lncRNA, is derived from an intron of the *SPRY4* gene and is predicted to contain several long hairpins in its secondary structure [59]. The lncRNA *HOTAIR* is also implicated in cancer [60], serving as a structural scaffold for protein complexes and possesses complex RNA structural motifs [61]. These structural motifs may act as distinct binding domains for protein complexes such as *PRC2* and *LSD1*, and serve in a manner of signal, guide or scaffold in different cellular contexts [62]. The lncRNA *Gas5* acts as both molecular decoy and signal to negatively regulate an effector. It has been examined that the lncRNA *SRA* has a complex structural organization, consisting of four domains, with a variety of secondary structure elements [53]. Moreover, the lncRNA structures may play critical roles in the interaction between lncRNA and other molecules such as chromatin-modifying complexes [8], chromatin [63] and miRNA [64]. All these suggest an important interplay between the lncRNA secondary structure and their biological functions.

The RNA secondary structure as well as the tertiary structure can be determined by experimental and computational methods. Because some large RNAs such as ribosomal RNAs and RNase P have already been successfully crystallized, the structural studies on lncRNAs will likely be possible in the near future. Because RNAs are extracted from cells and renatured in a buffer, the obtained structures in *in vitro* study may differ markedly from their *in vivo* forms. However, determining the RNA structures *in vitro* also has the important advantage of enabling studies on homogeneous populations of the targets and of using systems that are simpler than their *in vivo* counterparts. Comparing with computational methods, experimental methods can give a more reliable result, but with a higher experimental cost. On the other hand, computational methods can give large-scale investigation of lncRNA secondary structures with a low cost despite the high false-positive rate. For instance, in Volders *et al.* [65], the secondary structures of 21 488 human lncRNAs are predicted by the software RNAfold, and displayed via the graphics interchange format (.gif) in web browsers. In Rfam [66], the structure information for regions of higher conservation within the lncRNA transcripts is provided. The predicted results may provide clues in lncRNA studies, giving guidance to future experimental design. Still, a comprehensive whole-genome investigation of lncRNA secondary structures is lacking for any metazoan.

Recently, experimental techniques based on high-throughput sequencing have been developed to probe the RNA structures, such as SHAPE [67], parallel analysis of RNA structure (PARS) [68, 69] and FragSeq [70], which have enabled genome-wide measurements of paired and unpaired regions in the RNA secondary structures, and may shed a new light on lncRNA secondary structure analysis. Specifically, Li *et al.* [71] used a high-throughput, sequencing-based, structure-mapping approach to identify the paired (double-stranded RNA) and unpaired (single-stranded RNA) components of the *Drosophila melanogaster* and *Caenorhabditis elegans* transcriptomes, providing a global assessment of RNA folding in animals. Kertesz *et al.* [68] described a novel strategy termed PARS based on deep sequencing fragments of RNAs, and applied to profile the secondary structures of the mRNAs of the budding yeast *Saccharomyces cerevisiae*, and obtained structural profiles for over 3000 distinct transcripts. These initial studies indicate high-throughput sequencing-based methods as an effective and efficient approach for investigating RNA (lncRNAs included) secondary structures on a global scale. Related works have been reviewed in Mortimer *et al*. [72]. Another recent work [73] has also provided a comprehensive structure map of human coding and ncRNAs. However, like most existing experimental methods, high-throughput sequencing suffers from the disadvantage that it can only be used to assess the RNA structure *in vitro.* Obtained structures *in vitro* may differ markedly from their *in vivo* forms. Indeed, a fraction of the probed RNA secondary structures do not resemble the biologically functional state in many regions [9]. Thus, the methods based on high-throughput sequencing may not be as accurate as we can expect, especially for larger structured RNAs with long-range tertiary interactions. Nonetheless, it should be acknowledged that the advent of increasingly cheap high-throughput sequencing technologies make it possible to perform genome-wide investigation of the lncRNA secondary structures with a higher precision in comparison with direct computational prediction methods. Furthermore, genome-wide high-throughput sequencing structural data can be used to constrain folding algorithms and improve their accuracy, as previously shown for specific RNAs [74, 75]. Therefore, this huge catalog of structural sequencing data can provide us an opportunity to exploit these data collectively as a whole, especially when the lncRNA secondary structures are also considered.

## The function annotation of lncRNAs

From the previous discussion, it is noted that increasing evidence has been accumulated for the critical roles played by lncRNAs. However, when comparing to mRNAs, lncRNAs are generally expressed in a more tissue-specific manner [24, 25]. They also show lower expression level [24, 25, 38, 76], and higher expression variability across cell lines and tissues [25]. That is, the expression of lncRNAs may be regulated by subtle molecular mechanisms, but the lncRNAs themselves may function as a regulator in molecules. In this section, we will discuss the molecular mechanisms of several lncRNAs, and the current approaches devoted to lncRNA function annotation.

As a fact, the molecular mechanisms of most lncRNAs remain largely unknown. However, some clues have been provided recently by well-known examples. First, lncRNAs are found to be implicated in gene regulation through a variety of mechanisms such as epigenetic modifications of DNA, alternative splicing, posttranscriptional gene regulation and mRNA stability and translation [77–79]. Moreover, it is found that lncRNAs can regulate the expression of protein-coding genes, positively or negatively, and in cis or in trans [80]. For example, lncRNA *Kcnq1ot1* can regulate epigenetic gene silencing in an imprinted gene cluster in cis [81]. It is known that *Kcnq1ot1* specifically interacts with nearby genes in embryonic tissues causing transcriptional gene silencing. Another example is the lncRNA AK143260, termed *Braveheart* (*Bvht*), which acts in a trans manner and specifically promotes activation of a core gene regulatory network to direct cardiovascular lineage commitment [82]. In the recent two studies [24, 25], both cis-acting and trans-acting co-expression between lncRNAs and mRNAs have been observed. Second, lncRNAs are involved in cellular processes including proliferation, migration, apoptosis and development [83, 84], also in maintaining pluripotency [84, 85]. Based on these molecular features, lncRNAs can be categorized into different groups [33], such as signal, guide, scaffold and decoy. For example, *KCNQ1ot1*, *Air* and *Xist* are illustrated as signals of active silencing at their respective genomic locations, and others as guide, scaffold and decoy in [86].

Moreover, a complex interaction network exists between lncRNAs and other molecules such as miRNA, protein complex and other regulatory elements. Modular mechanisms have been proposed and ascribed to lncRNAs [87], providing an emerging model whereby lncRNAs may achieve regulatory specificity by assembling diverse combinations of proteins, and possibly with RNA and DNA interactions. For instance, a muscle-specific lncRNA, *linc-MD1*, could interact with two specific miRNAs, *miR-133* and *miR-135*, and promote muscle differentiation by acting as a competing endogenous RNA in mouse and human myoblasts [88]. The interactions between lncRNAs and other molecules are then exploited in other computational or experimental studies. For example, in Khalil *et al.* [8], the associations between lincRNAs and the polycomb repressive complex (PRC) 2 are studied, about 20% of 3300 lincRNAs expressed in various cell types are bound by PRC2 [8]. Accumulating associations between lncRNAs and other molecules are also predicted by computational methods or verified by experimental means [63, 89].

**Figure 1:**
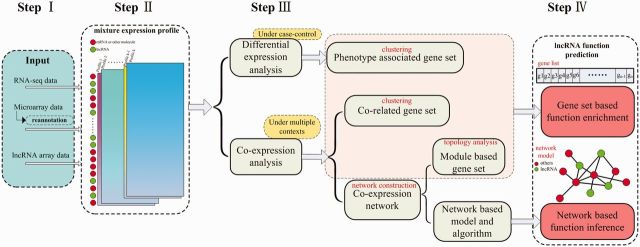
A framework of computational methods for lncRNA function prediction. (A colour version of this figure is available online at: http://bfg.oxfordjournals.org)

With the accumulating lncRNAs, there is a critical need to functionally annotate these lncRNAs. However, it is still a challenging task. First, undocumented structural features and weak conservation in their primary sequences for lncRNAs make it difficult to make inferences based on comparison. Second, there is a lack of a reliable network model on the relationships between lncRNAs and other molecules. Third and importantly, experimental validation of lncRNA functions is still expensive, labor-intensive and time-consuming. Fourth, subtle properties between the sequences, spatio-temporal and tissue-specific expression of lncRNAs, make them dynamic and elusive, increasing the difficulty. Nonetheless, pioneer works have been conducted. These works on lncRNA function annotations can be classified into two approaches, experimental and computational [90]. A framework of the computational methods is described in Figure 1. As for the input data, most of these methods are mainly based on the expression data for lncRNAs. One source of expression data is based on the RNA-seq sequencing. It can provide a comprehensive quantitative measure of the transcribed molecules in various samples. This includes the expression information of both lncRNAs and other RNA molecules. Another source is from the microarray data, which can be re-annotated based on further analysis because some of the probes are mapped to lncRNAs. A third source is based on the lncRNA array data with the probes specifically designed for lncRNAs. After obtaining the input data, in the second step, the mixture expression profiles for lncRNAs and mRNAs (or other molecules) are constructed. In the third step, differential expression analysis and co-expression analysis can be performed. The former is usually treated as case control, such as between the normal and the disease states [91]. The genes with differential expression profiles are then clustered into different gene sets, whereas the genes with similar expression profile are clustered into one gene set. The co-expression network between lncRNAs and other molecules can also be constructed based on the co-expression analysis. In co-expression network, different network modules are detected and the genes in one module are considered as a gene set. Models and algorithms can be designed and exploited based on the co-expression network. In the fourth step, strategies are employed to functionally annotate the lncRNAs. One strategy is based on the gene sets. For each gene set, function enrichment analysis is performed and the enriched function terms can be assigned to the un-annotated lncRNAs in the set. An example of this strategy can be found in Guttman *et al*. [26, 84]. Another strategy is based on a network model and uses specific algorithms. Algorithms are developed to infer the candidate functions of the lncRNAs in the network model. For example, lncRNA functions are predicted based on the network strategy in [90, 92, 93]. A global function predictor *lnc-GFP* was also developed by our group [90], which can effectively perform large-scale function prediction for lncRNAs. In this method, coding–noncoding co-expression data were integrated with protein interaction data to construct the bicolored network, on which a global method based on the information flow was designed to infer probable functions for as much lncRNAs as possible. Furthermore, the *lnc-GFP* was integrated into the webserver called ncFANs [94], which was developed to functionally annotate lncRNAs online.

## The databases for lncRNAs

Advances in transcriptome arrays and deep sequencing have given rise to fast accumulation of large data sets of lncRNAs. LncRNA transcripts and related information have recently been gathered in databases dedicated to lncRNA research. In this section, we summarize the content of general and specialized databases on lncRNAs. A recent work [33] has given a comprehensive report on the description and comparative evaluation of the resources and the computational tools, particularly of lncRNA databases. Here, we categorize the lncRNA databases into two main groups, the annotation databases and the interaction databases, in addition to other specific databases. The details are shown in Table 2 .

**Table 2:** The databases for lncRNAs

	Database	Web site	Reference
Annotation databases	NONCODE v 4.0	http://www.bioinfo.org/noncode/	[3]
	lncRNAdb	http://www.lncrnadb.org/	[95]
	LNCipedia	http://www.lncipedia.org	[65]
	lncRNome	http://genome.igib.res.in/lncRNome/	[96]
	fRNAdb	http://www.ncrna.org/frnadb	[97]
	lncRNAtor	http://lncrnator.ewha.ac.kr/	[98]
	lncRNAMap	http://lncrnamap.mbc.nctu.edu.tw/php/	[99]
	PLncDB	http://chualab.rockefeller.edu/gbrowse2/homepage.html	[100]
Interaction databases	ChIPBase	http://deepbase.sysu.edu.cn/chipbase/	[101]
	NPInter	http://www.bioinfo.org.cn/NPInter/	[102]
	miRcode	http://www.mircode.org/	[103]
	DIANA-LncBase	http://www.microrna.gr/LncBase	[64]
	StarBase v2.0	http://starbase.sysu.edu.cn/mirLncRNA.php	[104]
	lncRNA2Target	http://mlg.hit.edu.cn/lncrna2target/	[105]
	lncRNADisease	http://cmbi.bjmu.edu.cn/lncrnadisease	[106]
Specific databases	lnCeDB	http://gyanxet-beta.com/lncedb/	[107]
	NRED	http://nred.matticklab.com/cgi-bin/ncrnadb.pl	[108]
	Linc2go	http://www.bioinfo.tsinghua.edu.cn/∼liuke/Linc2GO/index.html	[109]
	lncRNASNP	http://bioinfo.life.hust.edu.cn/lncRNASNP/	[110]

Regarding the annotation databases, information such as the sequences, expressions, available secondary structures, related function and other internal information of lncRNAs are given. Other than comprehensive databases such as GenBank [111], FANTOM [112], HinvDB [113], GeneCards [114] and the ENCODE project [1] include annotated lncRNAs and publish their updated issue regularly. The general knowledge-based databases such as NONCODE [3], lncRNome [96] and LNCipedia [65] can offer a good compromise between coverage and depth of annotations. All these annotations can provide useful information in the understanding of lncRNAs. NONCODE is an integrated knowledge database dedicated to ncRNAs (excluding tRNAs and rRNAs). Particularly in its fourth version, the number of lncRNAs has increased sharply from 73 327 to 210 831 (accessed on 27 November 2013). Another example is lncRNAdb [95], it provides comprehensive annotations of eukaryotic lncRNAs, and enables the systematic compilation and updating of increasing data describing the expression profiles, the molecular features and related functions of individual lncRNA. It is designed especially for the list of lncRNAs that have been shown to have, or to be associated with, biological functions in eukaryotes, as well as messenger RNAs that have regulatory roles. Some annotation databases are developed for a specific organism, such as PLncDB [100] for *Arabidopsis.* Other annotation databases have also documented some interactions between lncRNAs and other molecules, such as fRNAdb [97], lncRNAtor [98] and lncRNome [96].

As for the interaction-based databases, ChIPBase [101], NPInter [102], miRcode [103], lncRNA2Target [105] and others are included. These databases deposit the relationships between lncRNAs and other molecules, which are retrieved by experimental methods or computational prediction. Several databases give insights into the potential regulatory roles of human lncRNAs and their interaction with miRNAs (Starbase v2.0 [104]), as well as sRNAs (LncRNAMap [99]), and proteins (LncRNAtor). LncRNAtor also provides information on co-expression between mRNAs and lncRNAs in various tissues. In addition, DIANA-LncBase [64] is focused on regulatory associations between miRNAs and lncRNAs, in which both experimental and computational interactions are included. Moreover, databases such as lncRNADisease [106] and lnCeDB [107] are also included in this group, which focus on the functional or logical relationship between lncRNAs and others. Detail comparisons of the lncRNA databases are available in the review of Fritah *et al.* [33].

Apart from the resources categorized above, some databases are designed for specific purpose and also listed in Table 2, such as NRED [108] for expression of ncRNAs, Linc2go [109] associating lncRNAs with gene ontology (GO) terms and lncRNASNP [110] including SNPs in the lncRNA regions. All these resources can be helpful for lncRNA research, especially for deep computational analysis of the lncRNA data.

It should be noted that these databases are important in delineating the transcript functional relationships. However, substantial divergence exists in the content and specific annotations among these resources [33] that researchers should be considered carefully.

## Conclusion

In summary, enormous progress has been made toward comprehensive annotation on thousands of lncRNAs with respect to their primary sequences, the structural features and their related functions. The mechanistic underpinnings of a few well-studied examples suggest that many of these transcripts might participate in important and diverse biological processes and human diseases. Current research is exploring how lncRNAs may participate in these cellular activities. To this end, expanding experimental techniques together with computational algorithms can provide important valuable insights.

With respect to the sequence level of lncRNAs, most studies focus on the comparison with mRNAs and the negative description of lncRNAs such as splicing pattern, 5′cap, poly A tail and properties related to ‘limited protein coding ability’. Hitherto, there is no general positive definition of lncRNA, despite advances in defining some of its subtypes and motifs embedded in the lncRNA sequences. With respect to the structure level of lncRNAs, components discovered in the lncRNA secondary structures are of great value for further analysis, especially based on high-throughput sequencing technologies. With respect to the function level of lncRNAs, increasing evidence has indicated important roles of lncRNAs in biological processes and diseases, even though the molecular mechanisms for most lncRNAs remain unknown. Nonetheless, the lncRNA expression data and the interactions between lncRNAs and other molecules may provide valuable important clues into the lncRNA functional mechanisms. In short, the coming advance in the study of lncRNAs, especially at a large genome-wide scale, poses an exciting opportunity to investigate the lncRNA function in the future.

**Key points**

- Many basic features in lncRNA sequences are found to be similar to that of mRNAs, even though the components in the lncRNA sequences encode a limited protein-coding ability, indicated using coding potential tools and other methods.
- Many secondary structural components in lncRNAs can be identified, as well as their related functional roles. High-throughput sequencing-based methods may shed light on probing the lncRNA secondary structures using a combination of computational prediction methods.
- While a few lncRNAs have been demonstrated to play key roles in various biological processes, the functional mechanisms of many lncRNAs remain poorly understood. Computational methods can be employed to predict probable functions of lncRNAs, mainly based on gene expression data for lncRNAs and others.
- The databases for lncRNAs can be categorized into two groups, the first group based on annotations, and the second group based on the relationships between lncRNAs and other molecules. Data from these resources can be used for further data-mining of important functional patterns of lncRNAs.

## References

[1] Consortium EP An integrated encyclopedia of DNA elements in the human genome. Nature 2012;489(7414):57–74. 22955616 10.1038/nature11247 PMC3439153

[2] Lander ES Linton LM Birren B Initial sequencing and analysis of the human genome. Nature 2001;409(6822):860–921. 11237011 10.1038/35057062

[3] Xie C Yuan J Li H NONCODEv4: exploring the world of long non-coding RNA genes. Nucleic Acids Res 2014;42(D1):D98–103. 24285305 10.1093/nar/gkt1222 PMC3965073

[4] Carninci P Kasukawa T Katayama S The transcriptional landscape of the mammalian genome. Science 2005;309(5740):1559–63. 16141072 10.1126/science.1112014

[5] Lau NC Seto AG Kim J Characterization of the piRNA complex from rat testes. Science 2006;313(5785):363–7. 16778019 10.1126/science.1130164

[6] David R Small RNAs: miRNA machinery disposal. Nat Rev Mol Cell Biol 2012;14(1):4–5. 10.1038/nrm3493 23212473

[7] Wilusz JE Sunwoo H Spector DL Long noncoding RNAs: functional surprises from the RNA world. Genes Dev 2009;23(13):1494–504. 19571179 10.1101/gad.1800909 PMC3152381

[8] Khalil AM Guttman M Huarte M Many human large intergenic noncoding RNAs associate with chromatin-modifying complexes and affect gene expression. Proc Natl Acad Sci USA 2009;106(28):11667–72. 19571010 10.1073/pnas.0904715106 PMC2704857

[9] Ulitsky I Bartel DP lincRNAs: genomics, evolution, and mechanisms. Cell 2013;154(1):26–46. 23827673 10.1016/j.cell.2013.06.020 PMC3924787

[10] Struhl K Transcriptional noise and the fidelity of initiation by RNA polymerase II. Nat Struct Mol Biol 2007;14(2):103–5. 17277804 10.1038/nsmb0207-103

[11] Shearwin KE Callen BP Egan JB Transcriptional interference–a crash course. TRENDS Genet 2005;21(6):339–45. 15922833 10.1016/j.tig.2005.04.009 PMC2941638

[12] Penny GD Kay GF Sheardown SA Requirement for Xist in X chromosome inactivation. Nature 1996;379(6561):131–7. 8538762 10.1038/379131a0

[13] Ponting CP Oliver PL Reik W Evolution and functions of long noncoding RNAs. Cell 2009;136(4):629–41. 19239885 10.1016/j.cell.2009.02.006

[14] Pauli A Rinn JL Schier AF Non-coding RNAs as regulators of embryogenesis. Nat Rev Genet 2011;12(2):136–49. 21245830 10.1038/nrg2904 PMC4081495

[15] Rinn JL Chang HY Genome regulation by long noncoding RNAs. Annu Rev Biochem 2012;81:145–66. 22663078 10.1146/annurev-biochem-051410-092902 PMC3858397

[16] Mitra SA Mitra AP Triche TJ A central role for long non-coding RNA in cancer. Front Genet 2012;3:17. 22363342 10.3389/fgene.2012.00017 PMC3279698

[17] Gibb EA Brown CJ Lam WL The functional role of long non-coding RNA in human carcinomas. Mol Cancer 2011;10(1):38–55. 21489289 10.1186/1476-4598-10-38 PMC3098824

[18] Bhan A Mandal SS Long noncoding RNAs: emerging stars in gene regulation, epigenetics and human disease. ChemMedChem 2014;9:1932–56. 24677606 10.1002/cmdc.201300534

[19] Zhi H Ning S Li X A novel reannotation strategy for dissecting DNA methylation patterns of human long intergenic non-coding RNAs in cancers. Nucleic Acids Res 2014;42:8258–70. 25013169 10.1093/nar/gku575 PMC4117791

[20] Du Z Fei T Verhaak RG Integrative genomic analyses reveal clinically relevant long noncoding RNAs in human cancer. Nat Struct Mol Biol 2013;20(7):908–13. 23728290 10.1038/nsmb.2591 PMC3702647

[21] Wapinski O Chang HY Long noncoding RNAs and human disease. Trends Cell Biol 2011;21(6):354–61. 21550244 10.1016/j.tcb.2011.04.001

[22] Tsai M-C Spitale RC Chang HY Long intergenic noncoding RNAs: new links in cancer progression. Cancer Res 2011;71(1):3–7. 21199792 10.1158/0008-5472.CAN-10-2483 PMC3057914

[23] Kung JT Colognori D Lee JT Long noncoding RNAs: past, present, and future. Genetics 2013;193(3):651–69. 23463798 10.1534/genetics.112.146704 PMC3583990

[24] Cabili MN Trapnell C Goff L Integrative annotation of human large intergenic noncoding RNAs reveals global properties and specific subclasses. Genes Dev 2011;25(18):1915–27. 21890647 10.1101/gad.17446611 PMC3185964

[25] Derrien T Johnson R Bussotti G The GENCODE v7 catalog of human long noncoding RNAs: analysis of their gene structure, evolution, and expression. Genome Res 2012;22(9):1775–89. 22955988 10.1101/gr.132159.111 PMC3431493

[26] Guttman M Amit I Garber M Chromatin signature reveals over a thousand highly conserved large non-coding RNAs in mammals. Nature 2009;458(7235):223–27. 19182780 10.1038/nature07672 PMC2754849

[27] Pagano A Castelnuovo M Tortelli F New small nuclear RNA gene-like transcriptional units as sources of regulatory transcripts. PLoS Genet 2007;3(2):e1. 17274687 10.1371/journal.pgen.0030001 PMC1790723

[28] Wang J Zhang J Zheng H Mouse transcriptome: neutral evolution of ‘non-coding’complementary DNAs. Nature 2004;431(7010):757. 15495343

[29] Ponjavic J Ponting CP Lunter G Functionality or transcriptional noise? Evidence for selection within long noncoding RNAs. Genome Res 2007;17(5):556–65. 17387145 10.1101/gr.6036807 PMC1855172

[30] Ørom UA Derrien T Beringer M Long noncoding RNAs with enhancer-like function in human cells. Cell 2010;143(1):46–58. 20887892 10.1016/j.cell.2010.09.001 PMC4108080

[31] Kutter C Watt S Stefflova K Rapid turnover of long noncoding RNAs and the evolution of gene expression. PLoS Genet 2012;8(7):e1002841. 22844254 10.1371/journal.pgen.1002841 PMC3406015

[32] Pang KC Frith MC Mattick JS Rapid evolution of noncoding RNAs: lack of conservation does not mean lack of function. Trends Genet 2006;22(1):1–5. 16290135 10.1016/j.tig.2005.10.003

[33] Fritah S Niclou SP Azuaje F Databases for lncRNAs: a comparative evaluation of emerging tools. RNA 2014;20(11):1655–65. 25323317 10.1261/rna.044040.113 PMC4201818

[34] Brannan CI Dees EC Ingram RS The product of the H19 gene may function as an RNA. Mol Cell Biol 1990;10(1):28–36. 1688465 10.1128/mcb.10.1.28-36.1990 PMC360709

[35] Okazaki Y Furuno M Kasukawa T Analysis of the mouse transcriptome based on functional annotation of 60 770 full-length cDNAs. Nature 2002;420(6915):563–73. 12466851 10.1038/nature01266

[36] Kapranov P Drenkow J Cheng J Examples of the complex architecture of the human transcriptome revealed by RACE and high-density tiling arrays. Genome Res 2005;15(7):987–97. 15998911 10.1101/gr.3455305 PMC1172043

[37] Nordström KJ Mirza MA Almén MS Critical evaluation of the FANTOM3 non-coding RNA transcripts. Genomics 2009;94(3):169–76. 19505569 10.1016/j.ygeno.2009.05.012

[38] Ravasi T Suzuki H Pang KC Experimental validation of the regulated expression of large numbers of non-coding RNAs from the mouse genome. Genome Res 2006;16(1):11–19. 16344565 10.1101/gr.4200206 PMC1356124

[39] Pruitt KD Tatusova T Brown GR NCBI Reference Sequences (RefSeq): current status, new features and genome annotation policy. Nucleic Acids Res 2012;40(D1):D130–5. 22121212 10.1093/nar/gkr1079 PMC3245008

[40] Grabherr MG Haas BJ Yassour M Full-length transcriptome assembly from RNA-Seq data without a reference genome. Nat Biotechnol 2011;29(7):644–52. 21572440 10.1038/nbt.1883 PMC3571712

[41] Trapnell C Williams BA Pertea G Transcript assembly and quantification by RNA-Seq reveals unannotated transcripts and isoform switching during cell differentiation. Nat Biotechnol 2010;28(5):511–15. 20436464 10.1038/nbt.1621 PMC3146043

[42] Dinger ME Pang KC Mercer TR Differentiating protein-coding and noncoding RNA: challenges and ambiguities. PLoS Comput Biol 2008;4(11):e1000176. 19043537 10.1371/journal.pcbi.1000176 PMC2518207

[43] Brockdorff N Ashworth A Kay GF The product of the mouse *Xist* gene is a 15 kb inactive X-specific transcript containing no conserved ORF and located in the nucleus. Cell 1992;71(3):515–26. 1423610 10.1016/0092-8674(92)90519-i

[44] Lin MF Jungreis I Kellis M PhyloCSF: a comparative genomics method to distinguish protein coding and non-coding regions. Bioinformatics 2011;27(13):i275–82. 21685081 10.1093/bioinformatics/btr209 PMC3117341

[45] Lin MF Carlson JW Crosby MA Revisiting the protein-coding gene catalog of Drosophila melanogaster using 12 fly genomes. Genome Res 2007;17(12):1823–36. 17989253 10.1101/gr.6679507 PMC2099591

[46] Kong L Zhang Y Ye Z-Q CPC: assess the protein-coding potential of transcripts using sequence features and support vector machine. Nucleic Acids Res 2007;35(suppl 2):W345–9. 17631615 10.1093/nar/gkm391 PMC1933232

[47] Wang L Park HJ Dasari S CPAT: Coding-Potential Assessment Tool using an alignment-free logistic regression model. Nucleic Acids Res 2013;41:e74. 23335781 10.1093/nar/gkt006 PMC3616698

[48] Sun L Luo H Bu D Utilizing sequence intrinsic composition to classify protein-coding and long non-coding transcripts. Nucleic Acids Res 2013:41:e166. 23892401 10.1093/nar/gkt646 PMC3783192

[49] Liu J Gough J Rost B Distinguishing protein-coding from non-coding RNAs through support vector machines. PLoS Genet 2006;2(4):e29. 16683024 10.1371/journal.pgen.0020029 PMC1449884

[50] Bateman A Coin L Durbin R The Pfam protein families database. Nucleic Acids Res 2004;32(suppl 1):D138–41. 14681378 10.1093/nar/gkh121 PMC308855

[51] Blanco E Parra G Guigó R Using geneid to identify genes. Curr Protoc Bioinform 2007:**Chapter 4**;Unit 4.3. 10.1002/0471250953.bi0403s18 18428791

[52] Ingolia NT Lareau LF Weissman JS Ribosome profiling of mouse embryonic stem cells reveals the complexity and dynamics of mammalian proteomes. Cell 2011;147(4):789–802. 22056041 10.1016/j.cell.2011.10.002 PMC3225288

[53] Kino T Hurt DE Ichijo T Noncoding RNA gas5 is a growth arrest-and starvation-associated repressor of the glucocorticoid receptor. Sci Signal 2010;3(107):ra8. 20124551 10.1126/scisignal.2000568 PMC2819218

[54] Maenner S Blaud M Fouillen L 2-D structure of the A region of Xist RNA and its implication for PRC2 association. PLoS Biol 2010;8(1):e1000276. 20052282 10.1371/journal.pbio.1000276 PMC2796953

[55] Novikova IV Hennelly SP Sanbonmatsu KY Structural architecture of the human long non-coding RNA, steroid receptor RNA activator. Nucleic Acids Res 2012;40(11):5034–51. 22362738 10.1093/nar/gks071 PMC3367176

[56] Wilusz JE JnBaptiste CK Lu LY A triple helix stabilizes the 3′ ends of long noncoding RNAs that lack poly (A) tails. Genes Dev 2012;26(21):2392–407. 23073843 10.1101/gad.204438.112 PMC3489998

[57] Wilusz JE Freier SM Spector DL 3’ end processing of a long nuclear-retained noncoding RNA yields a tRNA-like cytoplasmic RNA. Cell 2008;135(5):919–32. 19041754 10.1016/j.cell.2008.10.012 PMC2722846

[58] Wilusz JE Spector DL An unexpected ending: noncanonical 3′ end processing mechanisms. RNA 2010;16(2):259–66. 20007330 10.1261/rna.1907510 PMC2811654

[59] Khaitan D Dinger ME Mazar J The melanoma‐upregulated long noncoding RNA SPRY4-IT1 modulates apoptosis and invasion. Cancer Res 2011;71(11):3852–62. 21558391 10.1158/0008-5472.CAN-10-4460

[60] Gupta RA Shah N Wang KC Long non-coding RNA HOTAIR reprograms chromatin state to promote cancer metastasis. Nature 2010;464(7291):1071–6. 20393566 10.1038/nature08975 PMC3049919

[61] Tsai M-C Manor O Wan Y Long noncoding RNA as modular scaffold of histone modification complexes. Science 2010;329(5992):689–93. 20616235 10.1126/science.1192002 PMC2967777

[62] He S Liu S Zhu H The sequence, structure and evolutionary features of HOTAIR in mammals. BMC Evol Biol 2011;11(1):102. 21496275 10.1186/1471-2148-11-102 PMC3103462

[63] Chu C Qu K Zhong FL Genomic maps of long noncoding RNA occupancy reveal principles of RNA-chromatin interactions. Mol Cell 2011;44:667–78. 21963238 10.1016/j.molcel.2011.08.027 PMC3249421

[64] Paraskevopoulou MD Georgakilas G Kostoulas N DIANA-LncBase: experimentally verified and computationally predicted microRNA targets on long non-coding RNAs. Nucleic Acids Res 2013;41(D1):D239–45. 23193281 10.1093/nar/gks1246 PMC3531175

[65] Volders P-J Helsens K Wang X LNCipedia: a database for annotated human lncRNA transcript sequences and structures. Nucleic Acids Res 2013;41(D1):D246–51. 23042674 10.1093/nar/gks915 PMC3531107

[66] Burge SW Daub J Eberhardt R Rfam 11.0: 10 years of RNA families. Nucleic Acids Res 2013;41:D226–32. 23125362 10.1093/nar/gks1005 PMC3531072

[67] Low JT Weeks KM SHAPE-directed RNA secondary structure prediction. Methods 2010;52(2):150–8. 20554050 10.1016/j.ymeth.2010.06.007 PMC2941709

[68] Kertesz M Wan Y Mazor E Genome-wide measurement of RNA secondary structure in yeast. Nature 2010;467(7311):103–7. 20811459 10.1038/nature09322 PMC3847670

[69] Wan Y Qu K Ouyang Z Genome-wide mapping of RNA structure using nuclease digestion and high-throughput sequencing. Nat Protoc 2013;8(5):849–69. 23558785 10.1038/nprot.2013.045

[70] Underwood JG Uzilov AV Katzman S FragSeq: transcriptome-wide RNA structure probing using high-throughput sequencing. Nat Methods 2010;7(12):995–1001. 21057495 10.1038/nmeth.1529 PMC3247016

[71] Li F Zheng Q Ryvkin P Dragomir I Global analysis of RNA secondary structure in two metazoans. Cell Rep 2012;1(1):69–82. 22832108 10.1016/j.celrep.2011.10.002

[72] Mortimer SA Kidwell MA Doudna JA Insights into RNA structure and function from genome-wide studies. Nat Rev Genet 2014;15:469–79. 24821474 10.1038/nrg3681

[73] Wan Y Qu K Zhang QC Landscape and variation of RNA secondary structure across the human transcriptome. Nature 2014;505(7485):706–9. 24476892 10.1038/nature12946 PMC3973747

[74] Watts JM Dang KK Gorelick RJ Architecture and secondary structure of an entire HIV-1 RNA genome. Nature 2009;460(7256):711–16. 19661910 10.1038/nature08237 PMC2724670

[75] Mathews DH Disney MD Childs JL Incorporating chemical modification constraints into a dynamic programming algorithm for prediction of RNA secondary structure. Proc Natl Acad Sci USA 2004;101(19):7287–92. 15123812 10.1073/pnas.0401799101 PMC409911

[76] Guttman M Garber M Levin JZ Ab initio reconstruction of cell type-specific transcriptomes in mouse reveals the conserved multi-exonic structure of lincRNAs. Nat Biotechnol 2010;28(5):503–10. 20436462 10.1038/nbt.1633 PMC2868100

[77] Mercer TR Mattick JS Structure and function of long noncoding RNAs in epigenetic regulation. Nat Struct Mol Biol 2013;20(3):300–307. 23463315 10.1038/nsmb.2480

[78] Tripathi V Ellis JD Shen Z The nuclear-retained noncoding RNA MALAT1 regulates alternative splicing by modulating SR splicing factor phosphorylation. Mol Cell 2010;39(6):925–38. 20797886 10.1016/j.molcel.2010.08.011 PMC4158944

[79] Yoon J-H Abdelmohsen K Srikantan S LincRNA-p21 suppresses target mRNA translation. Mol Cell 2012;47(4):648–55. 22841487 10.1016/j.molcel.2012.06.027 PMC3509343

[80] Mercer TR Dinger ME Mattick JS Long non-coding RNAs: insights into functions. Nat Rev Genet 2009;10(3):155–9. 19188922 10.1038/nrg2521

[81] Pandey RR Mondal T Mohammad F *Kcnq1ot1* Antisense Noncoding RNA Mediates Lineage-Specific Transcriptional Silencing through Chromatin-Level Regulation. Mol Cell 2008;32(2):232–46. 18951091 10.1016/j.molcel.2008.08.022

[82] Klattenhoff CA Scheuermann JC Surface LE *Braveheart*, a Long Noncoding RNA Required for Cardiovascular Lineage Commitment. Cell 2013;152(3):570–83. 23352431 10.1016/j.cell.2013.01.003 PMC3563769

[83] Hu W Yuan B Flygare J Long noncoding RNA-mediated anti-apoptotic activity in murine erythroid terminal differentiation. Genes Dev 2011;25(24):2573–8. 22155924 10.1101/gad.178780.111 PMC3248679

[84] Guttman M Donaghey J Carey BW lincRNAs act in the circuitry controlling pluripotency and differentiation. Nature 2011;477(7364):295–300. 21874018 10.1038/nature10398 PMC3175327

[85] Ng SY Johnson R Stanton LW Human long non‐coding RNAs promote pluripotency and neuronal differentiation by association with chromatin modifiers and transcription factors. EMBO J 2012;31(3):522–33. 22193719 10.1038/emboj.2011.459 PMC3273385

[86] Wang KC Chang HY Molecular mechanisms of long noncoding RNAs. Mol Cell 2011;43(6):904–14. 21925379 10.1016/j.molcel.2011.08.018 PMC3199020

[87] Guttman M Rinn JL Modular regulatory principles of large non-coding RNAs. Nature 2012;482(7385):339–46. 22337053 10.1038/nature10887 PMC4197003

[88] Cesana M Cacchiarelli D Legnini I A long noncoding RNA controls muscle differentiation by functioning as a competing endogenous RNA. Cell 2011;147(2):358–69. 22000014 10.1016/j.cell.2011.09.028 PMC3234495

[89] Wang Y Chen X Liu Z-P De novo prediction of RNA–protein interactions from sequence information. Mol Biosyst 2013;9(1):133–42. 23138266 10.1039/c2mb25292a

[90] Guo X Gao L Liao Q Long non-coding RNAs function annotation: a global prediction method based on bi-colored networks. Nucleic Acids Res 2013;41(2):e35. 23132350 10.1093/nar/gks967 PMC3554231

[91] Dong R Jia D Xue P Genome-Wide Analysis of Long Noncoding RNA (lncRNA) Expression in Hepatoblastoma Tissues. PloS One 2014;9(1):e85599. 24465615 10.1371/journal.pone.0085599 PMC3894996

[92] Liao Q Liu C Yuan X Large-scale prediction of long non-coding RNA functions in a coding–non-coding gene co-expression network. Nucleic Acids Res 2011;39(9):3864–78. 21247874 10.1093/nar/gkq1348 PMC3089475

[93] Cogill SB Wang L Co-expression Network Analysis of Human lncRNAs and Cancer Genes. Cancer Inf 2014;13(Suppl 5):49. 10.4137/CIN.S14070 PMC4218681 25392693

[94] Liao Q Xiao H Bu D ncFANs: a web server for functional annotation of long non-coding RNAs. Nucleic Acids Res 2011;39(suppl 2):W118–24. 21715382 10.1093/nar/gkr432 PMC3125796

[95] Quek XC Thomson DW Maag JL lncRNAdb v2. 0: expanding the reference database for functional long noncoding RNAs. Nucleic Acids Res 2015;43:D168–73. 25332394 10.1093/nar/gku988 PMC4384040

[96] Bhartiya D Pal K Ghosh S lncRNome: a comprehensive knowledgebase of human long noncoding RNAs. Database 2013;2013:bat034. 23846593 10.1093/database/bat034 PMC3708617

[97] Mituyama T Yamada K Hattori E The Functional RNA Database 3.0: databases to support mining and annotation of functional RNAs. Nucleic Acids Res 2009;37(suppl 1):D89–92. 18948287 10.1093/nar/gkn805 PMC2686472

[98] Park C Yu N Choi I lncRNAtor: a comprehensive resource for functional investigation of long noncoding RNAs. Bioinformatics 2014;30:2480–5. 24813212 10.1093/bioinformatics/btu325

[99] Chan W-L Huang H-D Chang J-G lncRNAMap: a map of putative regulatory functions in the long non-coding transcriptome. Comput Biol Chem 2014;50:41–9. 24525374 10.1016/j.compbiolchem.2014.01.003

[100] Jin J Liu J Wang H PLncDB: plant long non-coding RNA database. Bioinformatics 2013;29(8):1068–71. 23476021 10.1093/bioinformatics/btt107 PMC3624813

[101] Yang J-H Li J-H Jiang S ChIPBase: a database for decoding the transcriptional regulation of long non-coding RNA and microRNA genes from ChIP-Seq data. Nucleic Acids Res 2013;41(D1):D177–87. 23161675 10.1093/nar/gks1060 PMC3531181

[102] Yuan J Wu W Xie C NPInter v2.0: an updated database of ncRNA interactions. Nucleic Acids Res 2014;42(D1):D104–8. 24217916 10.1093/nar/gkt1057 PMC3965026

[103] Jeggari A Marks DS Larsson E miRcode: a map of putative microRNA target sites in the long non-coding transcriptome. Bioinformatics 2012;28(15):2062–3. 22718787 10.1093/bioinformatics/bts344 PMC3400968

[104] Li J-H Liu S Zhou H starBase v2.0: decoding miRNA-ceRNA, miRNA-ncRNA and protein–RNA interaction networks from large-scale CLIP-Seq data. Nucleic Acids Res 2014;42(D1):D92–7. 24297251 10.1093/nar/gkt1248 PMC3964941

[105] Jiang Q Wang J Wu X LncRNA2Target: a database for differentially expressed genes after lncRNA knockdown or overexpression. Nucleic Acids Res 2015;43(D1):D193–6. 25399422 10.1093/nar/gku1173 PMC4383967

[106] Chen G Wang Z Wang D LncRNADisease: a database for long-non-coding RNA-associated diseases. Nucleic Acids Res 2013;41(D1):D983–6. 23175614 10.1093/nar/gks1099 PMC3531173

[107] Das S Ghosal S Sen R lnCeDB: database of human long noncoding RNA acting as competing endogenous RNA. PloS One 2014;9(6):e98965. 24926662 10.1371/journal.pone.0098965 PMC4057149

[108] Dinger ME Pang KC Mercer TR NRED: a database of long noncoding RNA expression. Nucleic Acids Res 2009;37(suppl 1):D122–6. 18829717 10.1093/nar/gkn617 PMC2686506

[109] Liu K Yan Z Li Y Linc2GO: a human LincRNA function annotation resource based on ceRNA hypothesis. Bioinformatics 2013;29(17):2221–2. 23793747 10.1093/bioinformatics/btt361

[110] Gong J Liu W Zhang J lncRNASNP: a database of SNPs in lncRNAs and their potential functions in human and mouse. Nucleic Acids Res 2014;43:D181–6. 25332392 10.1093/nar/gku1000 PMC4383871

[111] Benson DA Cavanaugh M Clark K GenBank. Nucleic Acids Res 2013;41(D1):D36–42. 23193287 10.1093/nar/gks1195 PMC3531190

[112] Consortium TF A promoter-level mammalian expression atlas. Nature 2014;507(7493):462–70. 24670764 10.1038/nature13182 PMC4529748

[113] Takeda J Yamasaki C Murakami K H-InvDB in 2013: an omics study platform for human functional gene and transcript discovery. Nucleic Acids Res 2013:41:D915–9. 23197657 10.1093/nar/gks1245 PMC3531145

[114] Safran M Dalah I Alexander J GeneCards Version 3: the human gene integrator. Database 2010;2010:baq020. 20689021 10.1093/database/baq020 PMC2938269

